# Supplementary material for: Content-rich biological network constructed by mining PubMed abstracts
Source: BMC Bioinformatics. 2004 Oct 8;5:147. doi: 10.1186/1471-2105-5-147 (PMC528731; doi:10.1186/1471-2105-5-147)
Supplement: Additional File 2 — The original results of the above study (non-essential files are deleted to keep the file size under the limit set by BMC bioinformatics). [file 1471-2105-5-147-S2.bz2 › chilibotAdditionalFile2/dip10/80ID9033409E292/html/PAK3_RAC1.html]

 


 **PAK3** and **RAC1** 
  
Found 258 abstracts in PubMed, retrieved 10.  
 

 What does Google say? 
 PDF only 
| .edu only 

---

**Interactive relationship** (e.g. stimulation, inhibition, etc)

**Inhibitory relationship**- Expression of RhoGDI, a negative regulator of  **Rac1** , inhibited secretory responses while overexpression of effectors of  **Rac1** , notably, p21  [ **PAK3** ]  activated kinase Pak1 and actin depolymerization factor ADF promoted evoked secretion.  Ref: 12754309 J Physiol< MedlineTA>J Physiol,
**Neutral relationship**- p21  [ **PAK3** ]  activated protein kinases PAK are a family of serine threonine protein kinases which are activated by binding of the p21  [ **PAK3** ]  G proteins Cdc42 or Rac  [ **RAC1** ] .  Ref: 12853446 J Biol Chem< MedlineTA>J Biol Chem,
- The p21  [ **PAK3** ]  activated protein kinases Paks are serine threonine protein kinases activated by binding to Rho family small GTPases, Rac  [ **RAC1** ]  and Cdc42.  Ref: 12860998 J Biol ChemJ Biol Chem,

**Non-interactive relationship** (e.g. studied together, co-existance, homology, etc.)

- In brain, Shank forms a complex with beta PIX and beta PIX associated signaling molecules including p21  [ **PAK3** ]  associated kinase PAK, an effector kinase of  **Rac1**  Cdc42.  Ref: 12626503 J Biol Chem, 2003
- The p21  [ **PAK3** ]  activated kinases PAKs 1 3 are serine threonine protein kinases whose activity is stimulated by the binding of active Rac  [ **RAC1** ]  and Cdc42 GTPases.  Ref: 12676796 Annu Rev Biochem< MedlineTA>Annu Rev Biochem, 2003
- Based on upregulation of p21  [ **PAK3** ]   **rac1**  and MAPK p38alpha, activation of the MAPK pathway appears to be an early event in colonic carcinogenesis.  Ref: 12865273 Gut, 2003
- RESULTS Evaluation of gene expression profiles of normal versus adenomatous colonic crypts of six different patients revealed, in general, dysregulation of up to 11% of all analysed genes total number n=588 specifically, p21  [ **PAK3** ]   **rac1**  was upregulated in four of six patients, mitogen activated protein kinase MAPK p38alpha in three of six patients, and interferon gamma receptor in three of six patients.  Ref: 12865273 Gut, 2003
- Addition of PAR1 AP inhibited basal Rac  [ **RAC1** ]  activity as well as the phosphorylation of the Rac  [ **RAC1** ]  effector, p21  [ **PAK3** ]  activated kinase PAK.  Ref: 12871383 J Thromb Haemost, 2003
- 3 The Rac  [ **RAC1** ]  binding domain of p21  [ **PAK3** ]  activated kinase is a potent inhibitor of activation by the p47phox and amphiphile independent pathway while exerting a milder inhibitory effect on the canonical pathway.  Ref: 12875368 Inflammation, 2003
- Inhibition of p21  [ **PAK3** ]  activated kinases Paks, downstream effectors of  **Rac1** , inhibited  **Rac1**  Q61L induced MT growth and retrograde flow.  Ref: 12796474 J Cell Biol, 2003
- In these samples, upregulation of proliferation associated genes ras oncogene related p21  [ **PAK3** ]   **rac1**  and MAPK p38alpha as well as downregulation of apoptosis related genes FAST kinase and p53 most likely reflects specific alterations in adenomas with low grade dysplasia.  Ref: 12865273 Gut, 2003
